# Tissue Distribution and Depuration of the Extracted Hepatotoxic Cyanotoxin Microcystins in Crucian Carp (*Carassius carassius*) Intraperitoneally Injected at a Sublethal Dose

**DOI:** 10.1100/tsw.2008.101

**Published:** 2008-07-31

**Authors:** Hehua Lei, Ping Xie, Jun Chen, Gaodao Liang, Ting Yu, Yan Jiang

**Affiliations:** Donghu Experimental Station of Lake Ecosystems, State Key Laboratory of Freshwater Ecology and Biotechnology of China, Institute of Hydrobiology, The Chinese Academy of Sciences, Wuhan 430072, China

**Keywords:** microcystins, intraperitoneal injection, crucian carp, tissue distribution, depuration

## Abstract

An acute toxicity experiment was conducted by intraperitoneal injection with a sublethal dose of extracted microcystins (MCs), 50 μg MC-LR (where L = leucine and R = arginine) equivalent/kg body weight (BW), to examine tissue distribution and depuration of MCs in crucian carp (*Carassius carassius*). Liver to body weight ratio increased at 3, 12, 24, and 48 h postinjection compared with that at 0 h (*p* < 0.05). MC concentrations in various tissues and aquaria water were analyzed at 1, 3, 12, 24, 48, and 168 h postinjection using liquid chromatography coupled with mass spectrometry (LC-MS). The highest concentration of MCs (MC-RR + MC-LR) was found in blood, 2–270 ng/g dry weight (DW), followed by heart (3–100 ng/g DW) and kidney (13–88 ng/g DW). MC levels were relatively low in liver, gonad, intestine, spleen, and brain. MC contents in gills, gallbladder, and muscle were below the limit of detection. Significant negative correlation was present between MC-RR concentration in blood and that in kidney, confirming that blood was important in the transportation of MC-RR to kidney for excretion. Rapid accumulation and slow degradation of MCs were observed in gonad, liver, intestine, spleen, and brain. Only 0.07% of injected MCs were detected in liver. The recovery of MCs in liver of crucian carp seemed to be dose dependent.

## INTRODUCTION

Cyanobacterial blooms and the production of cyanotoxins represent a serious global problem[1]. Microcystins (MCs) are the most prevalent cyanotoxins, which are produced by members of cyanobacterial genera including *Microcystis*, *Oscillatoria*, *Anabaena*, *Nostoc*, *Anabaenopsis,* and *Hapalosiphon*[2]. Structurally, MCs are monocyclic heptapeptides that contain two L-amino acids, in which main structural variations are observed, and five D-amino acids[3]. So far, more than 80 structural variants of MCs are known, among which the most common and also the most extensively studied MCs are MC-LR and MC-RR (where L = leucine and R = arginine)[4,5].

The toxicity of MCs is mediated through inhibition of serine-threonine protein phosphatases 1 and 2A[6]. Fish can be exposed to MCs either during feeding or passively, when the toxins pass through the gills during breathing, and fish mortality is reported in ponds and lakes where toxic cyanobacterial blooms have collapsed[7,8]. Fish exposed to acutely toxic concentrations of MCs or bloom material showed damage in liver, kidney, gut, or gills; disturbances of the ion balance; changes in cardiac function; growth inhibition; and mortality[9].

Some field studies demonstrate that MCs can accumulate in fish tissues (especially in liver) and may be transferred farther up the food chain[10,11,12,13,14,15]. Information on tissue distribution of MCs in toxic experiments on fishes is still limited. Acute or subchronic toxic experiments have been conducted to study tissue distribution of MCs on cold-water carnivorous fishes, such as Atlantic salmon[16,17] and rainbow trout[18,19], and warm-water phytoplanktivorous fishes, such as *Tilapia rendalli*[20], silver carp[21], and bighead carp[22]. Recently, we studied the distribution and depuration of two common MCs (MC-RR and MC-LR) in various tissues of warm-water omnivorous crucian carp (*Carassius carassius*) via intraperitoneal injection with lethal dose of extracted MCs, 200 µg MC-LR equivalent/kg body weight (BW)[23].

In the present study, crucian carp were injected intraperitoneally with a sublethal dose of extracted MCs (50 µg MC-LR equivalent/kg BW) to examine tissue distribution and clearance of MCs. The results of the present study are compared with our previous study when crucian carp received a lethal dose of extracted MCs[23].

## EXPERIMENT

Crucian carp (*C. carassius*) with mean weight 265 ± 22.6 g (n = 105) were purchased from a local fish hatchery in Wuhan City (Hubei, China). Fish were acclimated for 2 weeks in seven aquaria (150 l, 15 fish per aquarium) containing dechlorinated tap water and fed with commercial crucian carp food at a rate of 2% BW/day. Feeding was terminated 2 days before initiation of the experiment and no food was supplied to the fish during the experimental period. Water temperature was controlled at 25 ± 1$^{o}$C, and dissolved oxygen was 6.8 ± 0.7 mg/l.

The cyanobacterial material used in this experiment was collected from surface blooms of Lake Dianchi (Yunnan, China). Freeze-dried crude algae were extracted three times with 5% acetic acid in water, purified with C18 reversed-phase cartridge[23], and finally suspended in distilled water (containing 34.1 µg/ml of MC-RR and 5.7 µg/ml of MC-LR). A dose of an approximately 1-ml suspension of extracted solution of MCs was injected intraperitoneally along the ventral midline into the peritoneum of fish, amounting to 150 µg/kg BW of MC-RR plus MC-LR. According to Lei et al.[23], this dose was equivalent to 50 µg/kg of purified MC-LR.

Fifteen test fish were collected for toxin analysis at 1, 3, 12, 24, 48, and 168 h postinjection. Fish without administration expressed as 0 h. All liver, kidney, spleen, intestine, brain, heart, gallbladder, gill, gonad, muscle, and blood on each sampling time were weighed, immediately frozen, and lyophilized for MC analysis. Extraction of MCs in fish tissues basically followed the method of Lei et al.[23].

Qualitative and quantitative analysis of MCs was performed using a Finnigan liquid chromatography-mass spectrometry (LC-MS) system (Thermo Electron, Waltham, MA, USA) comprising a Thermo Surveyor autosampler, a Surveyor mass spectrum pump, a Surveyor photodiode-array system, and a Finnigan LCQ-Advantage MAX ion trap mass spectrometer equipped with an electrospray ionization source (ESI). The instrument control, data processing, and analysis were conducted by using Xcalibur software (Ver 3, Thermo Electron). Separation was carried out under the reversed phase on Agilent Zorbax SB-C18 column (length, 100 mm; inner diameter, 2.1 mm; film thickness, 3.5 µm; Agilent Technologies, Santa Clara, CA, USA). The linear gradient program was as follows: 0 min 5% B, 0.5 min 30% B, 3 min 40% B, 6 min 70% B, 14.5 min 70% B, 14.6 min 5% B, 20 min 5% B. Sample injection volumes were typically 10 µl. MS was set to ESI+ mode and MS tuning and optimization were achieved by infusing MC-RR with ion of $[M+2H]^{2+}$ at *m/z* of 520. Quantification of MCs was achieved through total signal of MS/MS. Precursor ion was $[M+2H]^{2+}$ at *m/z* of 520 for MC-RR, while precursor ion was $[M+H]^{+}$ at *m/z* of 995.5 for MC-LR. Collision energy was 37% for both MC-RR and MC-LR. All the values present in the text were measured by ESI-LC/$MS^2$.

## RESULTS

No mortality was found during the experimental period. Table 1 shows the liver, kidney, and spleen to BW ratio throughout the experimental period. Liver to BW ratio increased at 3, 12, 24, and 48 h postinjection compared with that at 0 h ($p < 0.05$, Table 1). There were no significant changes in either kidney to BW ratio or spleen to BW ratio throughout the experimental period ($p > 0.05$, Table 1).

**TABLE 1**
**Liver to BW Ratio (L/BW), Kidney to BW Ratio (K/BW), and Spleen to BW Ratio (S/BW) of Crucian Carp (*C. carassius*) Throughout the Experimental Period (*n* = 15)**

| Time after Injection (h) | L/BW (%) | K/BW (%) | S/BW (%) |
|---|---|---|---|
| 0 | 1.97 ± 0.20 | 0.41 ± 0.07 | 0.32 ± 0.07 |
| 1 | 2.95 ± 0.85 | 0.40 ± 0.06 | 0.33 ± 0.12 |
| 3 | 3.26 ± 1.19* | 0.39 ± 0.06 | 0.33 ± 0.10 |
| 12 | 3.40 ± 1.45* | 0.40 ± 0.07 | 0.34 ± 0.06 |
| 24 | 4.16 ± 1.56* | 0.44 ± 0.06 | 0.34 ± 0.07 |
| 48 | 3.63 ± 0.59* | 0.47 ± 0.09 | 0.34 ± 0.10 |
| 168 | 3.31 ± 1.07 | 0.44 ± 0.09 | 0.37 ± 0.13 |

* The significance levels observed are $p < 0.05$ in comparison to 0 h.

At 0 h, no MCs were detected in the fish tissues and aquarium water. Fig. 1 shows the distribution of MCs in the tissues of crucian carp after injection. The highest concentration of MCs (MC-RR + MC-LR) was found in blood, 2–270 ng/g dry weight (DW), followed by heart (3–100 ng/g DW) and kidney (13–88 ng/g DW). MC levels were relatively low in liver, gonad, intestine, spleen, and brain. MC contents in gills, gallbladder, and muscle were below the limit of detection. According to the conversion method of Lei et al.[23], MC contents in fish tissues were converted to values as percentage of the injected MCs. There were 0.01–1.38% of injected MCs detected in blood, 0.01–0.07% in liver, and 0.04–0.57% in gonad (Table 2). Other tissues contained less than 0.05% of injected MCs. MC-RR content in blood was correlated significantly with that in heart ($r = 0.979$, $p < 0.01$), gonad ($r = 0.954$, $p < 0.01$), and kidney ($r = –0.821$, $p < 0.05$, Table 3).

The maximum MC concentration in the aquaria water was observed at 24 h postinjection, about 1.56% of injected MCs being detected. No significant correlations existed among MC-RR concentrations in the aquaria water and fish tissues ($p > 0.05$).

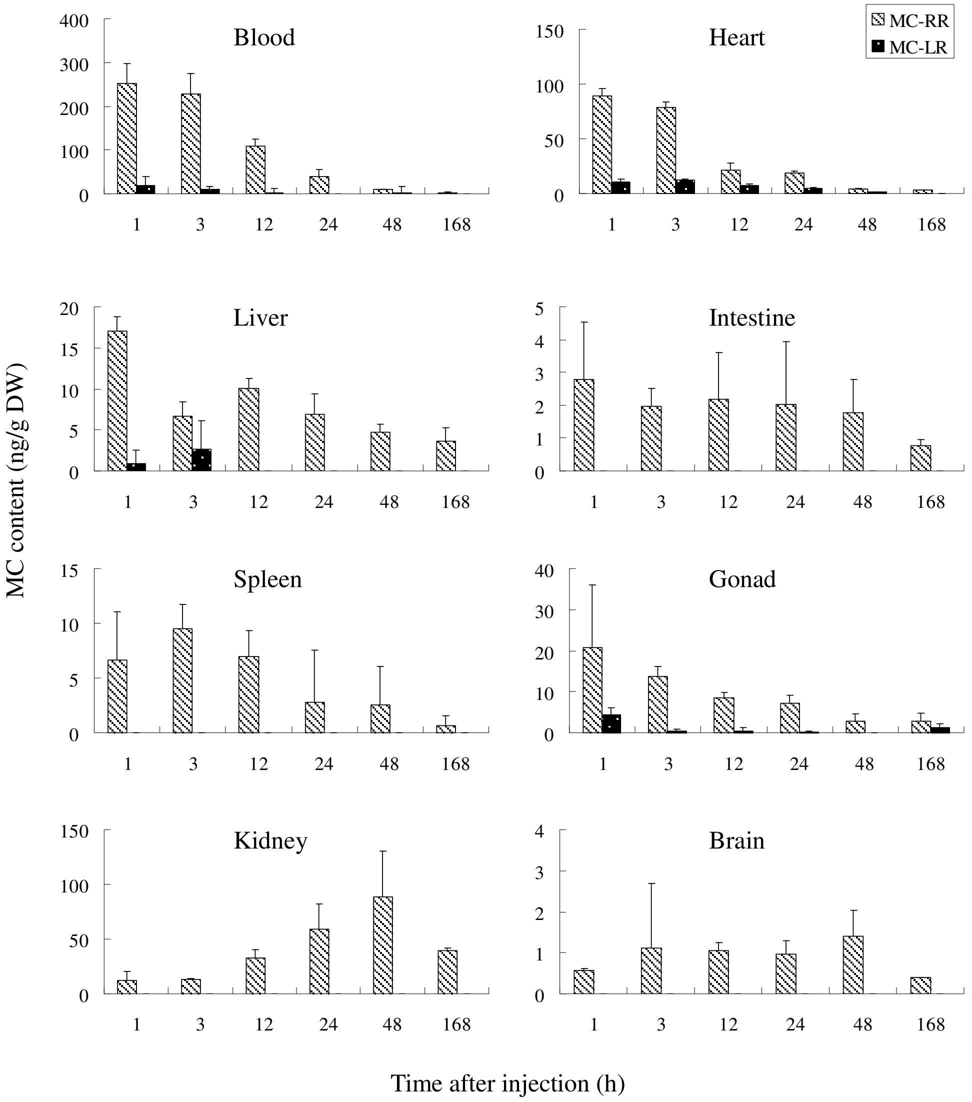

**FIGURE 1**. MC levels detected in crucian carp (*C. carassius*) tissues after intraperitoneal injection with 50 µg MC-LR equivalent/kg BW ($n$ = 3).

**TABLE 2**
**MC Contents in Blood, Liver, and Gonad of Crucian Carp (*C. carassius*) as Percentage of Injected MCs ($n$ = 3)**

| Time after Injection (h) | Blood (%) | Liver (%) | Gonad (%) |
|---|---|---|---|
| 1 | 1.38 ± 0.26 | 0.07 ± 0.03 | 0.57 ± 0.33 |
| 3 | 1.11 ± 0.21 | 0.07 ± 0.06 | 0.22 ± 0.07 |
| 12 | 0.46 ± 0.11 | 0.03 ± 0.00 | 0.15 ± 0.04 |
| 24 | 0.15 ± 0.07 | 0.02 ± 0.01 | 0.11 ± 0.04 |
| 48 | 0.09 ± 0.05 | 0.02 ± 0.00 | 0.04 ± 0.03 |
| 168 | 0.01 ± 0.01 | 0.01 ± 0.00 | 0.13 ± 0.08 |

**TABLE 3**
**Correlation among MC-RR Contents in Tissues of Crucian Carp (*C. carassius*)**

| | Blood | Heart | Liver | Intestine | Spleen | Gonad |
|---|---|---|---|---|---|---|
| Blood | | | | | | |
| Heart | 0.979** | | | | | |
| Liver | 0.749 | 0.712 | | | | |
| Intestine | 0.702 | 0.664 | 0.847* | | | |
| Spleen | 0.883* | 0.799 | 0.527 | 0.651 | | |
| Gonad | 0.954** | 0.958** | 0.878* | 0.781 | 0.738 | |
| Kidney | –0.821* | –0.783 | –0.575 | –0.309 | –0.691 | –0.770 |

Significant at $*p < 0.05$ and $**p < 0.01$ levels.

## DISCUSSION

In the present study, when crucian carp were injected intraperitoneally with a sublethal dose of extracted MCs, the liver to BW ratio increased in the fish (Table 1), although no intrahepatic hemorrhage and no mortality were found during the experimental period. Increase in liver weight is a characteristic toxic effect of MCs[24] and is reported in a number of animal models[25,26]. Kotak et al.[25] suggested that the increase in liver weight in rainbow trout exposed to MC-LR may be due to water retention in the liver since straw-colored fluid oozed from the fish livers when they were sectioned. The present study supports this suggestion since similar hydropic degeneration was also found in the liver of crucian carp treated with extracted MCs.

In the present study, the highest MC concentrations were found in blood (1.38% of injected dose) at 1 h. Significant negative correlation was also present between MC-RR concentration in blood and that in kidney. This is in good agreement with our prior study when crucian carp were injected with a lethal dose of extracted MCs, and confirms that blood was important in the transportation of MC-RR to the kidney for excretion[23]. MCs are eventually removed from the general circulation by renal or subsequent hepatic elimination, and kidneys are potentially exposed to greater concentrations of MCs when the toxins are not subjected to presystemic hepatic elimination[27]. Thus, high MC levels in kidneys induced renal damage as reported in both laboratory studies[25,28] and field studies[29], and further caused anemia in fish[30].

Relative high MC levels were present in the hearts of crucian carp in both the present study and our previous study[23]. Little is known about the effect of MCs on the fish heart. In brown trout alevins, crude cell extracts of *Microcystis* significantly increase heart rate, stroke volume, and cardiac output at environmentally relevant concentrations of cyanobacterial biomass equivalent to 5 μg/L MC-LR[31]. Liu et al.[32] found that hepatotoxicity and cardiotoxicity were the main lesions from MC-LR in loach larvae, based on ultrastructural alteration in hepatocytes and heart. MC exposure induced lesions in the heart and modification of heart rate, stroke volume, and cardiac output have also been reviewed in mammals[33,34]. LeClaire et al.[33] suggested that MC may potentially induce cardiopathy.

In the present study, the clearance of MCs in the gonad was slow. After 7 day's depuration, 22.8% of accumulated MCs were still in the gonad. Low clearance rate was also shown in our previous study; at the end of the experiment, 18.8% of accumulated MCs were present in the gonad of crucian carp[23]. These findings raise questions about the probable reproductive toxicity of MCs in fish that have been evidenced in mammals[35].

In the present study, rapid accumulation and slow degradation of MCs were also observed in liver, intestine, spleen, and brain, although MC levels in these tissues were relative low. Only 0.07% of injected

MCs were detected in the liver, which was much lower than that obtained in our previous study, 1.60% in the liver, when crucian carp received a lethal dose of extracted MCs[23]. The recovery of MCs in the liver of crucian carp seemed to be dose dependent. However, tritium-labeled MC-LR distribution in mice tissue at death or 6 h postinjection was similar for all doses (13–101 μg $^{3}$H-MC-LR/kg)[36]. It is necessary to note that results obtained in our study referred to free MCs in fish tissues, while tritium distribution in mice tissue included free MCs and MC metabolites. Assuming that 20% of injected MCs entered into the liver of fish weighting 250 g, i.e., 10 and 2.5 μg, MCs entered into the liver at a dose of 200 and 50 μg/kg MCs, respectively. If 2 μg of these MCs metabolized in the liver, only 0.5 μg (4% of injected dose) were free MCs at 50 μg/kg dose, while 8 μg (16%) were free MCs at 200 μg/kg dose. So the extremely low recovery of MCs in the present study is not surprising.

## ACKNOWLEDGMENTS

The authors would like to thank the College of Fishery, Huazhong Agricultural University, China, for their assistance in this experiment. This work was supported by a National Basic Research Program of China (973 Program) (Grant No. 2008CB418101) and by the National Natural Science Foundation of China (30700077).
